# Modelling of substrate access and substrate binding to cephalosporin acylases

**DOI:** 10.1038/s41598-019-48849-z

**Published:** 2019-08-27

**Authors:** Valerio Ferrario, Mona Fischer, Yushan Zhu, Jürgen Pleiss

**Affiliations:** 1 Institute of Biochemistry and Technical Biochemistry, University of Stuttgart, Allmandring 31, 70569 Stuttgart, Germany; 2 Department of Chemical Engineering, Tsinghua University, Beijing, 100084 China

**Keywords:** Computational models, Biocatalysis

## Abstract

Semisynthetic cephalosporins are widely used antibiotics currently produced by different chemical steps under harsh conditions, which results in a considerable amount of toxic waste. Biocatalytic synthesis by the cephalosporin acylase from *Pseudomonas sp*. strain N176 is a promising alternative. Despite intensive engineering of the enzyme, the catalytic activity is still too low for a commercially viable process. To identify the bottlenecks which limit the success of protein engineering efforts, a series of MD simulations was performed to study for two acylase variants (WT, M6) the access of the substrate cephalosporin C from the bulk to the active site and the stability of the enzyme-substrate complex. In both variants, cephalosporin C was binding to a non-productive substrate binding site (E86α, S369β, S460β) at the entrance to the binding pocket, preventing substrate access. A second non-productive binding site (G372β, W376β, L457β) was identified within the binding pocket, which competes with the active site for substrate binding. Noteworthy, substrate binding to the protein surface followed a Langmuir model resulting in binding constants K = 7.4 and 9.2 mM for WT and M6, respectively, which were similar to the experimentally determined Michaelis constants K_M_ = 11.0 and 8.1 mM, respectively.

## Introduction

**Figure 1:**
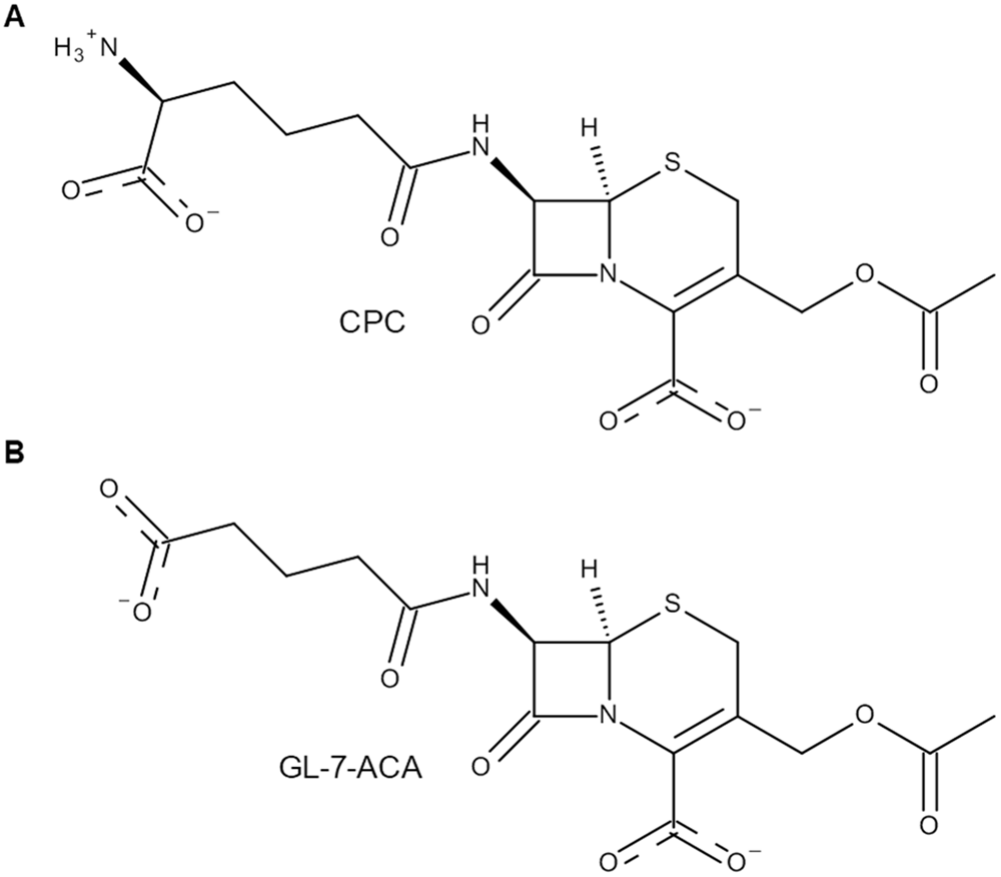
Structures of cephalosporin C (CPC) (**A**) and glutaryl-7-amino cephalosporanic acid (GL-7-ACA) (**B**).

Semisynthetic cephalosporins are widely used antibiotics to protect against extended-spectrum β-lactamase producing pathogens. Cephalosporin antibiotics are usually synthesized from 7-amino cephalosporanic acid (7-ACA) which is obtained by the hydrolysis of cephalosporin C (CPC) available from fermentation (Fig. 1A)^1^. Industrial approaches for cephalosporins production require different chemical steps under harsh reaction conditions which also result in the formation of toxic waste^2–5^. Thus, an enzymatic process able to efficiently catalyze the hydrolysis of CPC to 7-ACA would be a desirable alternative for the production of semisynthetic cephalosporins, reducing waste and the number of necessary process steps^6^. It has been demonstrated that glutaryl acylases (GAs), which use glutaryl-7-ACA (GL-7-ACA) as substrates (Fig. 1B), show a low hydrolytic activity toward CPC^7^. GAs are classified on the basis of their gene structures, molecular masses, and enzyme properties^8^. Out of five GA classes, enzymes from class I and III have low hydrolytic activity toward CPC, with an enzyme from class III, the GA from *Pseudomonas sp*. strain N176, having the highest activity, which corresponds to 4% of its hydrolytic activity toward GL-7ACA^8,9^. Therefore, GAs from class I and III are frequently called cephalosporin acylases (CAs). The structures of class I KAC-1 from *Pseudomonas diminuta* (PDB: 1FM2 and 1JVZ)^10,11^, CA from *Pseudomonas sp*. 130 (PDB: 1GK0 and 1GHD)^12,13^, CA from *Pseudomonas sp*. GK16 (PDB: 1OR0 and 1OQZ)^14^, and of class III CA from *Pseudomonas sp*. strain N176 (PDB: 4HSR and 4HST)^15^ were experimentally determined and compared. They share a similar structure, high sequence identity (>90%), and a similar substrate specificity^15^. From the structural point of view, CAs are α/β heterodimers resulting from a single folded precursor which undergoes autocatalytic cleavage to produce the mature enzyme^14,15^. Interestingly, the residue responsible for the autocatalytic process, the N-terminal serine of the β-chain, is also essential for the catalytic activity of the mature form of the enzyme. Thus, CAs are N-terminal hydrolases^10–15^. The buried substrate binding pocket is located at the interface between the two protein chains, and enzyme-substrate interactions were investigated using co-crystallized substrates^10–15^. Based on structural information, a catalytic mechanism of the class III enzyme from *Pseudomonas sp*. strain N176 was proposed^15^. Like other N-terminal hydrolases, the N-terminal amine group acts as a base to deprotonate the hydroxyl group of the same residue. Subsequently, the N-terminal Ser1β performs a nucleophilic attack to the carbonyl group of the substrate, resulting in the formation of a tetrahedral intermediate, which is stabilized by the oxyanion hole formed by the side chain of Asn242β and the backbone amino group of His70β. The reaction proceeds to CPC hydrolysis via the release of 7ACA and the subsequent nucleophilic attack of a water molecule (Fig. S1).

Because of its industrial potential for cephalosporin production, intensive research efforts have been devoted to improve the catalytic activity of the class III enzyme from *Pseudomonas sp*. strain N176. Random mutations led to the identification of mutant M31βF (further called WT) with a twofold increase of v_max_^1,16^. More recently, active site residues involved in CPC stabilization were targeted by mutagenesis approaches, resulting in the identification of seven hotspot positions (M165α, H57β, F58β, H70β, I176β, D177β, H178β)^15,17–19^. Notably, mutant M165αS/H57βS/H70βS resulted in a fourfold increase of v_max_ in comparison to WT. Further activity improvements were obtained by including mutations M31βF/F58βN/H70βS/I176βT to WT^20^. Even if protein stability is not an issue in respect to the commonly used experimental conditions, alternative approaches were devoted to improve enzyme evolvability, since stabilizing mutations are expected to compensate for loss of stability possibly caused by beneficial mutations for enzyme activity^21,22^. Thus, two stabilizing mutations were identified (L154βF/L180βF)^19^.

However, despite the profuse effort during the last 20 years and the promising results, the obtained catalytic activities are not yet sufficient to encourage 7-ACA manufacturers to shift to the single-step enzymatic conversion of CPC into 7-ACA at industrial level^1^. With all the approaches tested so far, it was not possible to increase the activity toward CPC by more than one order of magnitude. There seems to be a glass ceiling preventing substantial enhancement of catalytic activity. Engineering strategies have addressed so far only the optimization of interactions between the enzyme and the substrate in a productive binding pose close to its transition state. Such a design strategy misses two important bottlenecks that might limit catalytic activity: the presence of non-productive substrate binding poses (meaning binding poses not compatible with the catalytic mechanism) which compete with productive binding^23^, and the access of substrate from the bulk to the active site^24^. To address those possible limitations, molecular dynamics (MD) simulations were performed and two enzyme variants were compared: M31βF (WT) and M31βF/F58βN/H70βS/I176βT (M6)^20^. MD simulations were performed to analyze the enzyme-substrate interactions within the enzyme binding pocket and to investigate the diffusion of the substrate into the enzyme binding pocket, starting from experimental substrate concentrations. The simulations were analyzed to identify non-productive binding sites in the enzyme binding pocket and bottlenecks upon substrate access.

## Results

Two different series of molecular dynamics (MD) simulations were performed to compare the binding of the substrate cephalosporin C (CPC) to two variants of cephalosporin acylase (CA) from *Pseudomonas sp*. N176: wild type M31βF (WT) and M31βF/F58βN/H70βS/I176βT (M6). The access of CPC molecules to the protein binding pocket of CA variants was analyzed by simulations of CA in CPC solutions at 4 different concentrations to investigate possible concentration effects and to determine the concentration dependency of substrate binding. In a second series of simulations, the orientation and position of a CPC molecule in the substrate binding pocket of CA was modelled starting from an enzyme-substrate complex, where the CPC substrate was placed in a productive binding pose, corresponding to the Near Attack Conformation (NAC)^25–27^ (Fig. S1A). The analysis of the simulations was based on the distance *d*_*NAC*_ between the active site of CA and the carbonyl group of CPC (see Methods section).

### Simulation of substrate access: the free energy profile of CPC

The access and the interactions of CPC molecules with CA variants were modeled by unbiased MD simulations of a single CA molecule in CPC solutions at 4 different concentrations by adding 11, 20, 50, or 100 CPC molecules to the same volume. For each concentration, 5 independent simulations of 200 ns each were performed. Thus, each molecular system was sampled for 1 µs in total, and for all CPC molecules the distance *d*_*NAC*_ was measured every ps (200000 frames sampled for each simulation run). To ensure the stability of the system (i.e. no relevant conformational changes of the protein structure), the average RMSD of the 5 independent runs for the highest CPC concentration has been calculated for both WT and M6 backbones (Fig. S2). Assuming a Boltzmann distribution^28,29^, the free energy profile of CPC was calculated as difference between the negative logarithm of the number of CPC molecules with a given *d*_*NAC*_, counted for bins of 1 Å, and the respective reference states (Fig. S3). For each *d*_*NAC*_ bin, the free energy of the reference state was calculated from the negative logarithm of the number of CPC molecules at the respective concentration in the absence of the enzyme (eq. 4).

**Figure 2:**
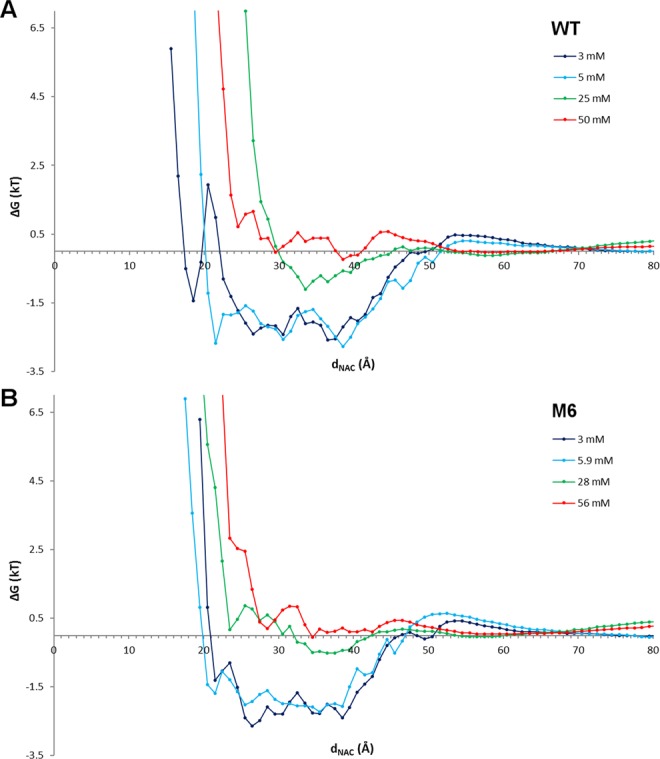
Free energy profile of CPC. Free energies (*ΔG* expressed in *kT*) calculated for WT (**A**) and M6 (**B**) as a function of the distance *d*_*NAC*_ at 4 bulk concentrations of CPC.

In bins at large distances (*d*_*NAC*_ > 60 Å), the number of CPC molecules in presence and in absence of the enzyme is equal, and results from the bulk concentration of CPC after equilibration. Thus, the bulk concentrations after equilibration were determined as 3.0, 5.0, 25.0, and 50.0 mM for WT, and 3.0, 5.9, 28.0, and 56.0 mM for M6. The free energy profiles of CPC obtained at the four concentrations were almost identical for the two enzyme variants (Fig. 2). At *d*_*NAC*_ > 60 Å, the binding potential was zero, because the enzyme has no influence on the CPC distribution. At 25 Å < *d*_*NAC*_ < 40 Å, the CPC molecules were bound to the enzyme surface. For the two lowest concentrations, the free energy of CPC was about −2 kT and was almost constant, meaning that CPC diffuses freely on the protein surface. At concentrations above 25 mM, the free energy of CPC was close to zero values in the region 30 Å < *d*_*NAC*_ < 40 Å, and increased to positive values at 25 Å < *d*_*NAC*_ < 30 Å indicating saturation of the protein surface close to the entrance to the binding pocket. At *d*_*NAC*_ < 25 Å, corresponding to CPC molecules at the entrance to the binding pocket, the free energy profile of CPC steeply increased, indicating the existence of a free energy barrier. No substrate molecule was observed at *d*_*NAC*_ < 18 Å, indicating that no CPC molecule were able to cross the free energy barrier at the entrance to the binding pocket within the simulation time.

**Figure 3:**
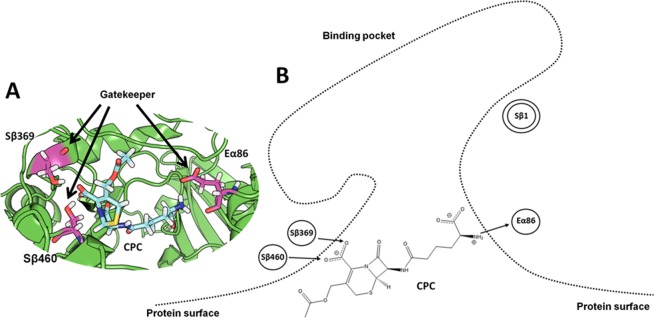
CPC at the entrance to the binding pocket. In the left part (**A**) the structural representation of CPC at the entrance to the binding pocket, CPC represented in cyan stick mode and the gatekeeper residues (S369β, S460β, E86α) in magenta stick mode. In the right part (**B**) the schematic representation: the catalytic serine (S1β) is labeled by a double circle, the gatekeeper residues (S369β, S460β, E86α) at the entrance to the binding pocket are indicated as circles; arrows indicate the electrostatic interactions between amino acid side chains and CPC.

Since the enzyme mutations are all located deeply in the binding pocket, no differences are expected at the surface. Therefore, the barrier preventing access of CPC to the enzyme binding pocket was identified by analyzing all the trajectory frames of the two CA variants where a CPC molecule was at 18 Å < *d*_*NAC*_ < 20 Å, resulting in a total of 60000 conformers. The different conformers of the CPC molecules were clustered based their RMSD (all atoms), after superimposition of the respective protein structures (using the Cα positions). The centroid structure of the largest cluster represented 95% of all conformers. A single CPC molecule was permanently bound close to the entrance to the binding pocket, interacting with three residues S369β, S460β, and E86α by electrostatic interactions (Fig. 3). This interaction network resulted in a CPC molecule oriented perpendicularly to the axis of the substrate access channel, thus blocking the entrance to other CPC molecules. At increasing CPC concentration, more CPC molecules were binding to the protein surface close to the entrance. However, none of them entered the substrate access channel, because they were blocked by a single CPC molecule specifically bound to the side chains of the gatekeeper residues S369β, S460β, and E86α. The interaction of CPC with the gatekeeper residues was stable during the simulation time: once a CPC molecule was bound to the gatekeeper residues, it did not leave the binding site during the simulation time.

### Binding affinity

**Figure 4:**
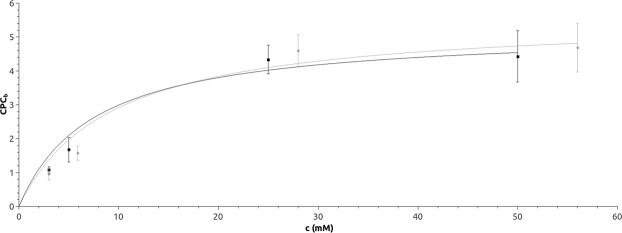
Langmuir model. The numbers of bound CPC molecules (*CPC*_*b*_) at *d*_*NAC*_ < 25 Å for different CPC concentrations c were fitted to a Langmuir model (WT: black line, M6: grey line).

At the experimental pH of 8.0^15–17,19,20^, the electrostatic potential (calculated by APBS)^30^ of the protein surface of both CA variants is mostly negative, except for a positive patch close to the entrance to the binding pocket (Fig. S4). At pH 8.0, CPC is negatively charged and therefore is expected to preferentially bind to the protein surface close to the entrance to the binding pocket (*d*_*NAC*_ ≈ 25 Å), as confirmed by the negative binding profiles at low CPC concentrations (Fig. 2). However, at higher CPC concentrations the free energy increased, indicating saturation of the binding sites on the protein surface closed to the entrance to the binding pocket. The concentration dependency of the number of CPC molecules binding to the protein surface close to the entrance to the binding pocket (*d*_*NAC*_ < 25 Å) followed a Langmuir model^31^ with a binding constant *K* = 7.4 ± 3.0 mM for WT and *K* = 9.2 ± 4.4 mM for M6 (Fig. 4). The simulated binding constants were similar to the experimentally determined values of the Michaelis constants *K*_*M*_ of 11 mM^16^ and 8.1 ± 0.6 mM^19^ for WT and M6, respectively.

### Simulation of the enzyme-substrate complex: productive and non-productive binding poses

**Figure 5:**
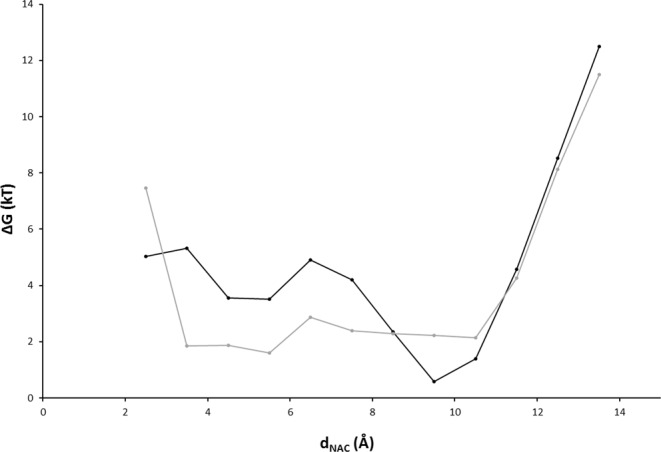
Analysis of conformations of the CPC substrate into the CA binding pocket. Probability calculated from *d*_*NAC*_ frequencies were plotted in logarithmic form and expressed in free energies as *kT* for WT (black) and M6 (grey).

Starting from a substrate bound into the active site at *d*_*NAC*_ = 2.2 Å (mean distance representing the NAC), the enzyme-substrate complexes of WT and M6 were simulated for 2 µs. For each enzyme, five independent simulations were performed, and the frequency of *d*_*NAC*_ was calculated from the last 1.8 µs of each simulation run (1800000 frames sampled for each simulation run). From the frequency, a free energy profile was calculated. Despite the long simulation time, no CPC molecule was observed at *d*_*NAC*_ > 14 Å (Fig. 5), indicating a free energy barrier blocking CPC from exiting the binding pocket in both enzyme variants. Below 12 Å, the free energy profiles of the two variants differed. While the profile of WT had a minimum at *d*_*NAC*_ = 9.5 Å and increased by 4 kT at *d*_*NAC*_ < 8 Å, the profile of M6 was almost constant at 3 Å < *d*_*NAC*_ < 11 Å.

**Figure 6:**
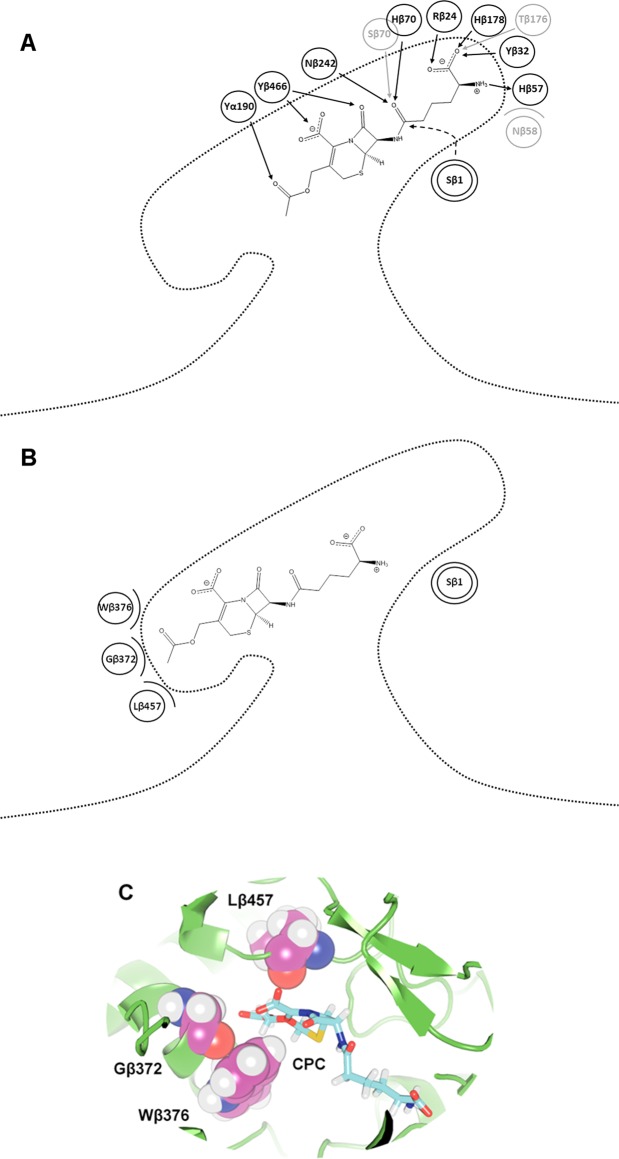
Representation of the CPC productive binding pose at *d*_*NAC*_ ≈ 3.5 Å (**A**) and the non-productive binding pose into the hydrophobic trap at *d*_*NAC*_ ≈ 9.5 Å (**B**,**C**). Residues interacting with CPC are indicated by circles. Additional mutations in M6 are highlighted in grey. The type of interaction is indicated by an arrow (hydrogen bonds) or by a curved line (steric interactions). The catalytic β1 serine is indicated by double circle and its nucleophilic attack by a dashed arrow.

In both enzymes, there were two major substrate binding poses: a productive binding pose at *d*_*NAC*_ ≈ 3.5 Å (closed to the Near Attack Conformation) and a non-productive binding pose at *d*_*NAC*_ ≈ 9.5 Å which is not compatible with the catalytic mechanism (Fig. 6). In WT, the conformations close to the Near Attack Conformation were stabilized by seven residues of the active site (R24β, Y32β, H57β, H70β, H178β, N242β, Y467β). The additional mutations in M6 contributed to a further stabilization: mutation H70βS improved binding of the oxyanion, mutation I176βT provided an extra electrostatic interaction, and mutation F58βN mediated a local side chain rearrangement resulting in an improved interaction of H57β with CPC (Fig. 6A). The non-productive binding pose at *d*_*NAC*_ = 9.5 Å was stabilized by three residues (Gβ372, Wβ376, Lβ457). This hydrophobic trap was identical in WT and M6 (Fig. 6B,C).

## Discussion

### Non-productive binding: the hydrophobic trap

Within the substrate binding pocket of the two CA variants, two sites competed for binding of the CPC molecule: the productive binding site close to the NAC (Fig. 6A) and the non-productive hydrophobic trap (Fig. 6B,C). There is growing evidence that in enzymes non-productive binding sites compete for substrate binding, especially in enzymes with large substrate binding pockets such as cytochrome P450 monooxygenases^32–35^. The relative binding affinity can be mediated by the reaction conditions. In aldolases, the population of a non-productive binding pose can increase at low pH^36^. Therefore, knowing the determinants of non-productive binding poses is crucial for rational protein design^37^, and improving the ratio between productive and non-productive binding might be the underlying principle of increasing catalytic activity upon directed evolution^38^. Blocking of non-productive binding sites could explain the activating effect of effector molecules like warfarin for CYP2C9^32^ or carboxylic acid for oleate hydratase^39,40^.

In both CA variants, the hydrophobic traps were identical, while the mutations Fβ58N/Hβ70S/Iβ176T^20^ in M6 improved the interaction with the substrate in the productive binding pose, thus shifting the equilibrium between the non-productive toward the productive binding pose. As a result, in M6 the substrate moved almost freely inside the binding pocket, while in WT it was trapped at *d*_*NAC*_ = 9.5 Å (Fig. 5). The hydrophobic trap is formed by Wβ379, Gβ372, and Lβ457, accommodating the acetyl moiety of the bulky CPC molecule. Thus, the presence of the hydrophobic trap reduced the frequency of the productive pose and, in addition, might contribute to competitive inhibition by substrate or by product. Removing the hydrophobic trap is expected to shift the equilibrium further toward the productive binding pose, consequently improving catalytic activity and preventing substrate or product inhibition by a negative design strategy^41^.

### Substrate access: the gatekeepers

In many enzymes, access of the substrate to the active site is limited by a barrier: gatekeeper residues at the entrance to the binding pocket^42^, a lid undergoing conformational transition between open and closed states^43,44^, or domain rearrangements which control substrate access^45^. While the latter two can be identified as distinct protein conformations under different crystallization conditions, the mobility of individual side chains or short gatekeeper loops might be hidden. Gatekeeper residues and narrow substrate access channels were identified by steered molecular dynamics simulations^46^ or by locally enhanced sampling techniques^47^. However, applying a biasing potential might obscure the underlying mechanisms and the preferred substrate pathway^48^. In contrast, performing unbiased MD simulations at realistic substrate concentrations is a promising modelling strategy to identify the molecular nature of barrier, such as the barrier in CA at 14 Å < *d*_*NAC*_ < 18 Å (Fig. 2). In the CA-CPC system, the barrier resulted from the binding of a CPC molecule to three gatekeeper residues (S369β, S460β, E86α) at the entrance to the binding pocket (Fig. 3). The perpendicular orientation of this specifically bound CPC molecule blocked the entrance to the binding pocket. Because the positively charged amino group of CPC interacts with a negatively charged side chain of the gatekeeper E86α (Fig. S5), the higher catalytic activity of CA from *Pseudomonas sp*. strain N176 toward GL-7-ACA^8,9^ might be explained by the missing amino group in the natural substrate GL-7-ACA.

As in all molecular dynamics simulations, the modelled interactions depend on the force field. However, the molecular properties underlying the enzyme-substrate interactions (shape and location of the hydrophobic trap, protonation state of the solvent-accessible gatekeeper residues and of the substrate) are described reliably by all force fields. Therefore, we expect a minor effect of the choice of the force field on the results.

### The molecular nature of K_M_

**Figure 7:**
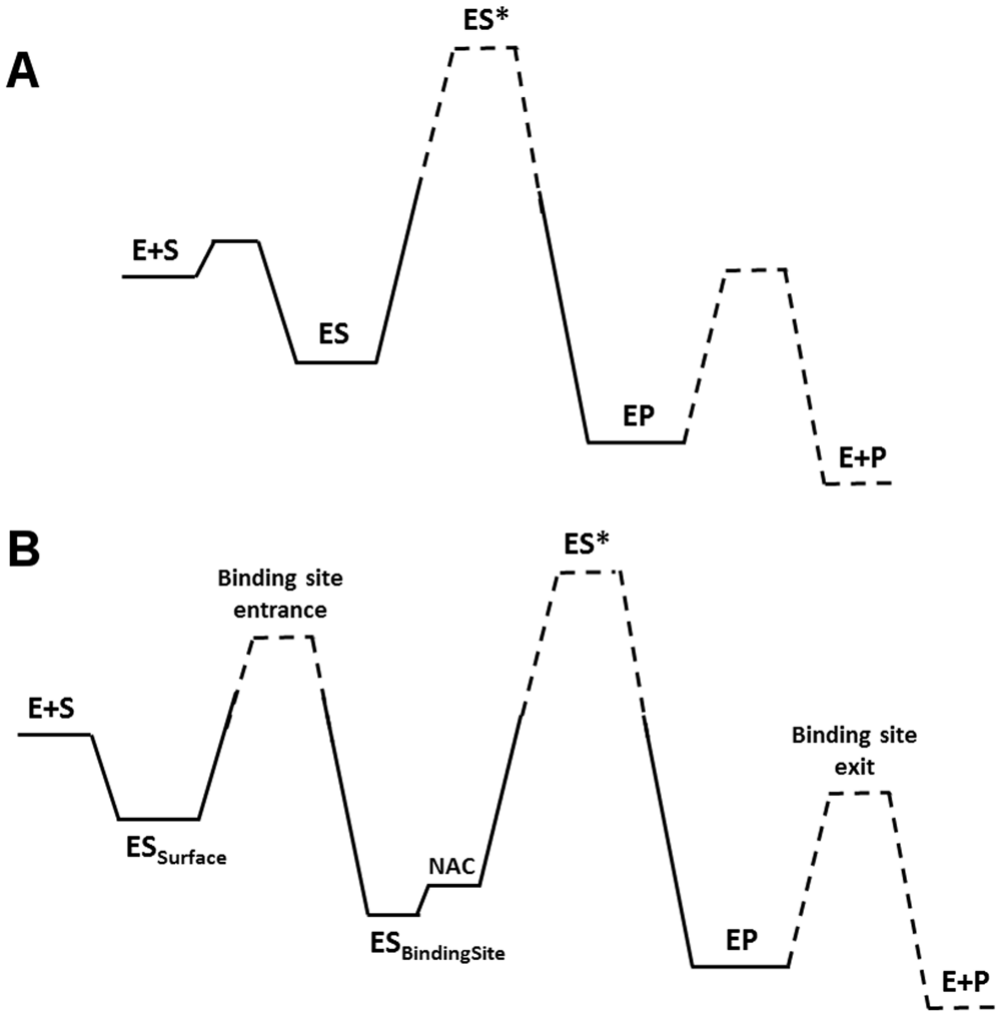
Schematic representation of the free energy profiles. The Michaelis-Menten model (**A**) and the proposed catalytic cycle of CA. (**B**) The states are represented as E + S for the free substrate, ES for the bound substrate state and ES* for the transition state of the chemical reaction. The cycle continue with the product bound to the enzyme (EP) and the free product after leaving the binding pocket (E + P). In CA, the bound state on the surface and within the binding pocket are distinct (ES_Surface_ and ES_BindingPocket_). Within the binding pocket, two sub-sites indicate the unproductive and the productive (NAC) binding pose.

The enzyme-catalyzed reaction is characterized by a transition from first order kinetics at low substrate concentration to zero-order kinetics at high substrate concentration. The transition to zero-order kinetics is characterized by the half-saturation concentration of the substrate, where the reaction rate is 50% of the maximum reaction rate. In the irreversible Michaelis-Menten model^49^, the experimentally observed half-saturation concentration is interpreted as the binding constant of the enzyme-substrate complex, assuming rate limitation upon the transition from the Michalis complex to the free product (Fig. 7A), and the reaction rate is described as:

1 $${v}_{0}=\frac{{k}_{cat}\cdot [E]\cdot [S]}{{K}_{M}+[S]}$$

The half-saturation concentration K_M_ includes binding and unbinding to the Michaelis complex and the chemical step. However, on a microscopic level, the Michaelis complex should not be interpreted as the Near Attack Conformation (NAC)^25–27^, because it comprises many binding events to productive and non-productive binding sites as well as conformational changes of the enzyme. Because each of these microscopic steps contributes to the observed saturation, we modeled the saturation of different sites by molecular dynamics simulation and compared the respective simulated half-saturation concentration to the experimentally determined K_M_. The computational procedure for studying binding to the protein surface was based on a series of 5 independent simulations of 200 ns for each simulated CPC concentration. Assuming ergodicity of simulated molecular system, the analysis of multiple independent simulations reliably links microstates with macroscopic properties^50^.

**Figure 8:**
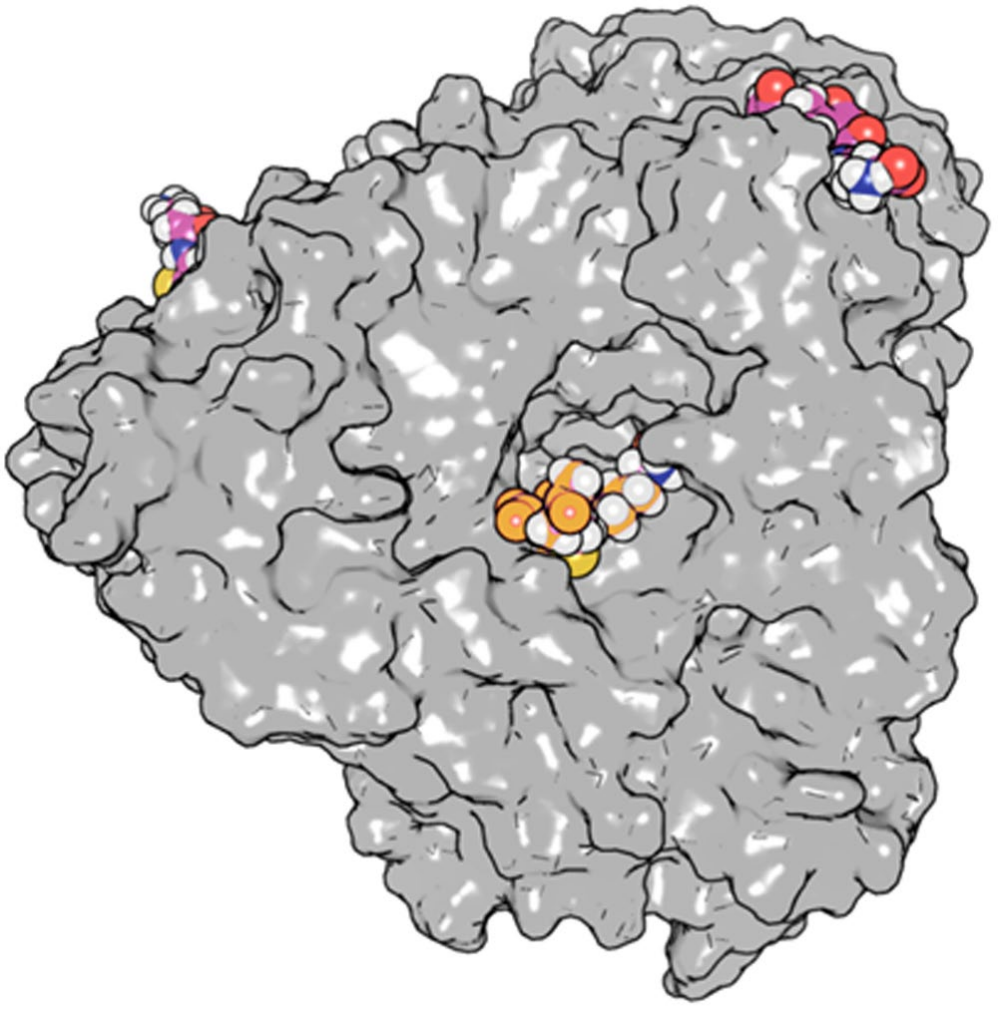
CPC molecules bound to the CA surface and to the gatekeeper residues. The protein surface is represented in grey while CPC molecules are represented in sphere mode. The CPC molecule in the center (orange) is bound to the gatekeeper residues at the entrance to the binding pocket.

In the catalytic cycle of CA, three distinct states were found (Fig. 7B): CPC in bulk (E + S), CPC bound to the protein surface (ES_Surface_), and CPC bound inside the binding pocket (ES_Binding pocket_). The substrate molecules bound to the protein surface (Fig. 8) diffused along the protein surface and frequently exchanged with the bulk state. No barrier was found between the bulk state and the surface-bound state, resulting in fast binding and unbinding of CPC to and from the protein surface during 200 ns of simulation time. However, there was a considerable barrier between the surface-bound state and the binding pocket.

We observed that the CPC binding sites on the protein surface were gradually saturated at increasing substrate concentration. The binding affinities obtained from molecular dynamics simulation (*K* = 7.4 ± 3.0 mM and 9.2 ± 4.4 mM for WT and mutant M6, respectively) were similar to the experimentally observed *K*_*M*_ values (11 and 8.1 mM, respectively)^17,20^. Therefore, it is intriguing to identify the experimentally observed saturation with CPC binding to the protein surface rather than into the substrate binding pocket, though we cannot exclude an additional contribution of the binding pocket. This interpretation is further supported by the experimental observation that mutations in the active site, though improving catalytic activity, had a negligible effect to *K*_*M*_^17,20^.

## Conclusions

The widely studied cephalosporin acylase from *Pseudomonas sp*. strain N176 was analyzed by a series of MD simulations in order to understand the glass ceiling limiting previous engineering efforts. Two distinct bottlenecks were identified: a hydrophobic trap in the binding pocket, which competes with productive binding to the active site, and gatekeeper residues on the protein surface, which restrict substrate access to the binding pocket. These functional hotspots have not been considered before, but they are promising targets for engineering and make a step forward toward the generation of a commercially viable biocatalyst with an improved turnover rate. Our systematic molecular dynamics simulations at different substrate concentrations also revealed a novel molecular interpretation of the experimentally determined Michaelis constant *K*_*M*_, which is mediated by binding of substrate to the protein surface rather than into the enzyme binding pocket.

## Methods

### Structures

The 3D crystal structure of cephalosporin acylase from *Pseudomonas sp*. N176 was retrieved from the Protein Data Bank (PDB entry 4HSR)^15,51^. This 2.13 Å resolution structure carries a single point mutation (M31βF) and it is referred as wild type (WT). The structure also contains the covalently bound ligand 5,5-dihydroxy-L-norvaline, which was removed. Mutant M31βF/F58βN/H70βS/I176βT^20^, referred here as M6, was constructed by the mutagenesis tool of PyMOL (The PyMOL Molecular Graphics System, Version 2.0 Schrödinger, LLC). The structure of CPC was taken from the Protein Data Bank (PDB entry 2VAV, ligand code CSC)^52^.

### Force fields, protonation states and system settings

Molecular dynamics (MD) simulations were performed using the software GROMACS version 5^53^ at constant pressure of 1 bar and at constant temperature of 310.15 K (NPT ensemble). The v-rescale and Berendsen algorithms were used for temperature and pressure coupling, respectively^54,55^. Electrostatic interactions were calculated by the smooth particle-mesh Ewald summation^56^. Water was simulated as SPC/E model^57^, while the CPC force field was derived by a RESP fit approach^58^. The RESP calculations were performed on the R.E.D. Server (RESP ESP charge Derive Server) where the software Firefly version 8 was used^59,60^. Partial charges were derived for the cephalosporin C core (Fig. S6) considering different possible conformations: all the low energy accessible conformations were computed using the software Confab setting 1 Å and 50 kcal/mol as structural and energy cut-offs^61^. The final CPC topology was obtained by using the tool MKTOP^62^ with standard OPLS atoms and using the partial charges coming from the RESP fit calculation together with those of the standard alanine OPLS definition (Fig. S6, CPC forcefield in supporting information). Such building block procedure was implemented to reuse the alanine OPLS definition. Since experimental activity measurements were performed at pH 8.0^15–17,19,20^, the same was considered for defining the protonation state of the simulated systems. The two acid moieties of CPC were considered as negatively charged, while the amino group was considered as positively charged, thus resulting in an overall CPC charge of −1. Protein force field definitions were obtained using the tool *pdb2gmx* of GROMACS 5. The pdb2pqr server was used to calculate the protonation state of each enzyme variant at pH 8.0^63^. For the two enzymes, the side chains of D/E and K/R were considered to be negatively and positively charged, respectively. Terminal residues were considered charged, except for the β1 serine which was defined as neutral, in agreement with the proposed catalytic mechanism (Fig. S1)^15^. The protonation states of the histidine residues are reported in Table S1.

### Simulation of the enzyme-substrate complex

Each modeled enzyme was simulated with a single CPC molecule manually placed into the binding pocket, with the substrate amide bond oriented to fit the stabilizing network in the catalytic mechanism (Fig. S1). The CPC orientation was adjusted to avoid steric clashes with the enzyme. The initial CPC orientation was identical for all the simulated systems. Interestingly, was not possible to obtain docked substrate poses in agreement with the catalytic mechanism by applying automated docking algorithms. Each enzyme-substrate complex system was then placed in the center of a cubic box of 1000 nm^3^. Each system was solvated using explicit SPC/E water^57,64^ and neutralized by adding the appropriate number of ions (Na^+^ or Cl^−^). Each system resulted in about 100000 atoms. For each enzyme-substrate complex considered, a series of five independent simulation runs was performed. Each system was minimized for 10000 steps, using a steepest descent algorithm and subsequently equilibrated for 10 ns. During the 10 ns equilibration, position restraints was applied to the protein heavy atoms and the CPC atoms (force constant 1000 kJ·mol^−1^·nm^−2^). The position restraints on CPC were gradually reduced during the equilibration (1000 kJ·mol^−1^·nm^−2^ for 4 ns, 500 kJ·mol^−1^·nm^−2^ for 3 ns, 300 kJ·mol^−1^·nm^−2^ for 3 ns). Subsequently, all the restraints were removed and each system was further equilibrated for 200 ns. After equilibration, each system was simulated for 1.8 µs. Thus, each enzyme variant in complex with CPC was simulated for a total time of 9 µs (5 independent runs of 1.8 µs each). Frames were saved every ps.

### Simulation of substrate access

Each modeled enzyme was simulated in a cubic box of 4096 nm^3^ and at four different CPC concentrations by adding a different number of substrate molecules (11, 20, 50, or 100 CPC molecules were randomly added using the GROMACS tool *gmx insert-molecules*). Each system was solvated using explicit SPC/E water^57,64^ and neutralized by adding the appropriate number of ions (Na^+^ or Cl^−^). Each system resulted in about 500000 atoms. Systems were minimized for 10000 steps using the steepest descent algorithm. For each CPC concentration, 5 independent simulations were performed. Each system was first equilibrated for 10 ns with position restraints applied to the protein heavy atoms and to the CPC molecules (force constant 1000 kJ·mol^−1^·nm^−2^). Subsequently, the restraints were removed, and the systems were further equilibrated for 50 ns. After the equilibration phase, each system was simulated and subsequently analyzed for 200 ns. Each enzyme variant in complex with CPC was simulated for a total time of 1 µs for each CPC concentration (5 independent runs of 200 ns each). Frames were saved every ps.

### NAC distance

**Figure 9:**
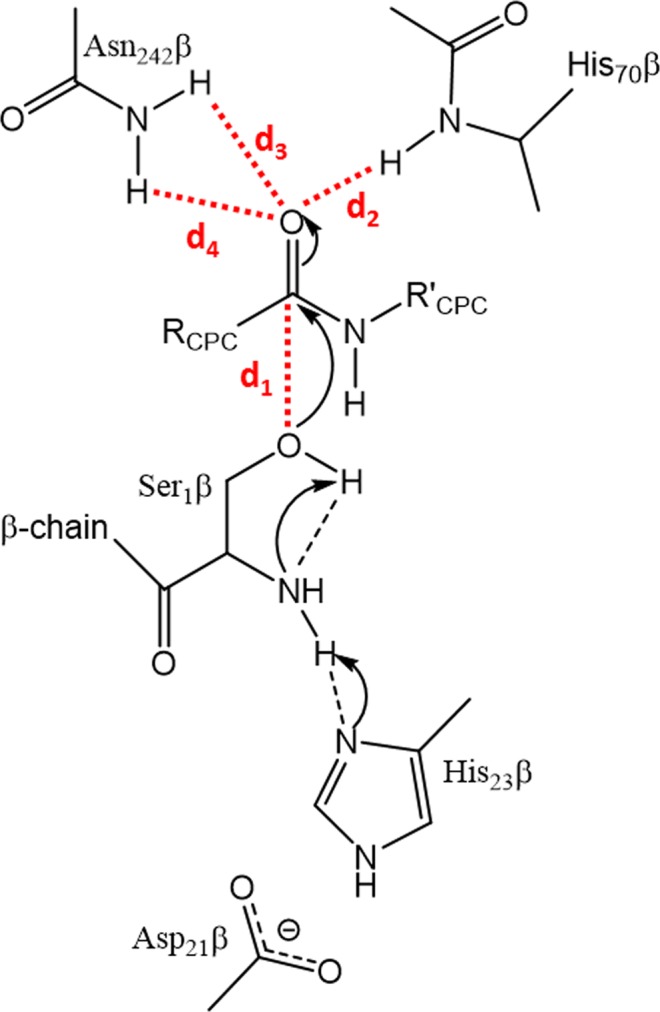
Schematic representation of the first step of the proposed catalytic mechanism. The substrate is assumed to bind in a ground state conformation which is closely related to the transition state of the chemical reaction: the Near Attack Conformation (NAC). The four distances used for calculating d_NAC_ are indicated in red and labeled.

According to the proposed catalytic mechanism, the substrate has to bind in a productive binding pose in its ground state. The latter closely resembles the transition state prior to the nucleophilic attack by the β1 serine side chain. This Near Attack Conformation (NAC)^25–27^ is characterized by four catalytically relevant distances (Fig. 9): between the hydroxyl oxygen of the catalytic β1 serine and the carbonyl carbon of the substrate (*d*_1_) and between the oxyanion hole residues and carbonyl oxygen of the substrate (*d*_2_, *d*_3_, *d*_4_).

A distance *d*_*NAC*_ was calculated as the root mean square of *d*_1_, *d*_2_, and the minimum of *d*_3_ and *d*_4_:

2 $${d}_{NAC}=\sqrt{\frac{{d}_{1}^{2}+{d}_{2}^{2}+{d}_{{\rm{\min }}(3,4)}^{2}}{3}}$$

*d*_*NAC*_ constitutes a reaction coordinate and was calculated at every ps and for every substrate molecule present in the simulation. In most simulations of the enzyme-substrate complex, *d*_*NAC*_ deviated from its initial value of 2.2 Å and varied in a range of 2.2 to 13 Å. A few simulations were discarded, because the value of *d*_*NAC*_ did not deviate from its initial value indicating kinetic trapping of CPC in its initial conformation.

### Free energy profile of CPC

The free energy profile of CPC was calculated as the logarithm of the ratio between the observed frequency of *d*_*NAC*_ in the presence of the enzyme and the calculated frequency at a given CPC concentration in the absence of the enzyme. *d*_*NAC*_ frequencies were summed up for all replicates and analyzed in bins of 1 Å.

The probability *p(i)* of having CPC molecule at bin *i* was obtained by dividing the number of substrate molecules found at bin *i* during the simulation by the total number of conformers analyzed:

3 $$p{(i)}_{enzyme}=\frac{{N}_{i}}{\#\,conformers}$$

where *N*_*i*_ represents the number of substrate molecules within a given bin (bins of 1 Å in *d*_*NAC*_) and *# conformers* indicates the total number of sampled conformers.

In a substrate solution at concentration *c* (in the absence of any enzyme), the number of substrate molecules *N*_*i*_ in a layer of thickness of *δb* = 1 Å at a distance a_*i*_ from the center is:

4 $$N{(i)}_{withoutenzyme}=\frac{4}{3}\pi ({({a}_{i}+\delta b)}^{3}-{{a}_{i}}^{3})\cdot c\cdot {N}_{0}$$

with Avogadro constant N_A_ = 6.022·10^23^ mol^−1^.

By considering the simulated system in a thermal equilibrium at temperature T, assuming a Boltzmann distribution, the probability of finding the system in a given state is related to its free energy^28,29^. Thus, the effect of the enzyme can be expressed as a free energy difference *ΔG* for each bin, and the free energy profile of CPC as a function of *d*_*NAC*_ is calculated as:

5 $$\frac{{\rm{\Delta }}{\rm{G}}}{kT}=-ln\,\frac{p{(i)}_{enzyme}}{N{(i)}_{withoutenzyme}}$$

At large distances (*d*_*NAC*_ > 60 Å), the enzyme does not interact with the substrate, and the free energy profile of CPC approaches 0. Therefore, the bulk concentrations *c* of the molecular systems after equilibration were obtained by fitting *p(i)*_*enzyme*_ and *N(i)*_*without enzyme*_ at *d*_*NAC*_ > 60 Å (Fig. S3).

### Conformational sampling

Conformational sampling of substrate poses was performed by isolating all the CPC molecules within a given *d*_*NAC*_ range. Therefore, all molecules except for the protein and the selected CPC molecules were discarded. The Cα atoms of the protein were used for superimposition of the selected conformers. Finally, the CPC molecules were clustered based on their RMSD using the *gmx cluster* of the GROMACS package and considering all the CPC atoms.

### Electrostatic properties

The electrostatic potential at the protein surface of the wild type enzyme (WT) was analyzed by the PyMol plugin for the APBS tool (Adaptive Poisson-Boltzmann Solver)^30^. Results were visualized on the protein structures using a range from −1 (red) to + 1 (blue).

### Binding affinity

A substrate molecule (CPC) was defined as bound to the protein surface close to the entrance to the binding pocket, if its center of mass was within 5 Å from any protein atom within 25 Å from the hydroxyl oxygen of β1 serine. The affinity of CPC for the enzyme was determined by fitting a Langmuir binding model^65^, assuming non-cooperative binding to a limited number of identical binding sites. The number of bound substrate molecules *CPC*_*b*_ was determined by counting (GROMACS tool *gmx trjorder*) the number of CPC molecules bound to the protein and by averaging over the simulation runs at the same substrate concentration. Standard errors were calculated by considering standard deviations from each simulation run and by error propagation during the averaging procedure. Finally, *CPC*_*b*_ was fitted with the CPC bulk concentration *c* by a Langmuir model^31,65^:

6 $$CP{C}_{b}=\frac{CP{C}_{b}^{MAX}\cdot c}{K+c}$$

where *CPC*_*b*_^*MAX*^ represents saturation (the maximum number of substrate molecules bound to the enzyme) and *K* the binding constant.

### Data deposition

The force field has been deposited as supplementary material.

## Supplementary information

Supplementary figures and tables
