# Supplementary material for: Modelling of substrate access and substrate binding to cephalosporin acylases
Source: Sci Rep. 2019 Aug 27;9:12402. doi: 10.1038/s41598-019-48849-z (PMC6712217; doi:10.1038/s41598-019-48849-z)
Supplement: Supplementary file 1 — Supplementary figures and tables [file 41598_2019_48849_MOESM1_ESM.docx]

Supporting information

**Modelling of substrate access and substrate binding to cephalosporin acylases**

Valerio Ferrario,^a^ Mona Fischer,^a^ Yushan Zhu,^b^ Jürgen Pleiss^a,^*

^a^ Institute of Biochemistry and Technical Biochemistry, University of Stuttgart, Allmandring 31, 70569 Stuttgart, Germany

E-mail: Juergen.Pleiss@itb.uni-stuttgart.de

^b^ Department of Chemical Engineering, Tsinghua University, Beijing 100084, China


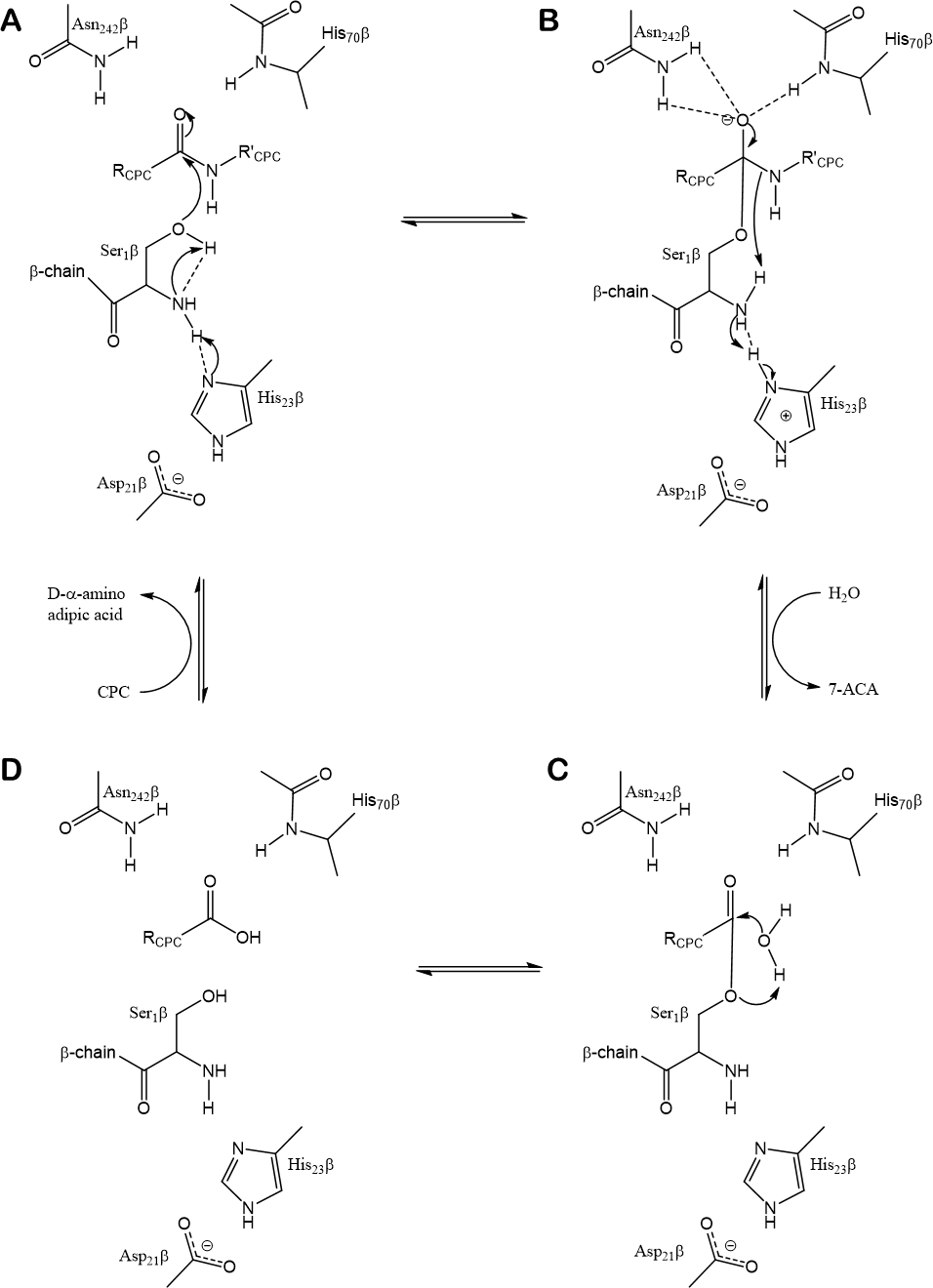


**Figure S1** Catalytic mechanism of cephalosporin C acylase from *Pseudomonas sp.* strain N176: nucleophilic attack bySer1β (A), tetrahedral intermediate state (B) , acyl enzyme and water nucleophilic attack (C), release of the product D-α-amino adipic acid (D).





**Figure S2.** RMSD calculation performed for WT (black line) and M6 (red line) using the backbone atoms. For each protein, the resulting RMSD represents an average of the 5 independent simulation runs of the systems with the highest CPC concentration.


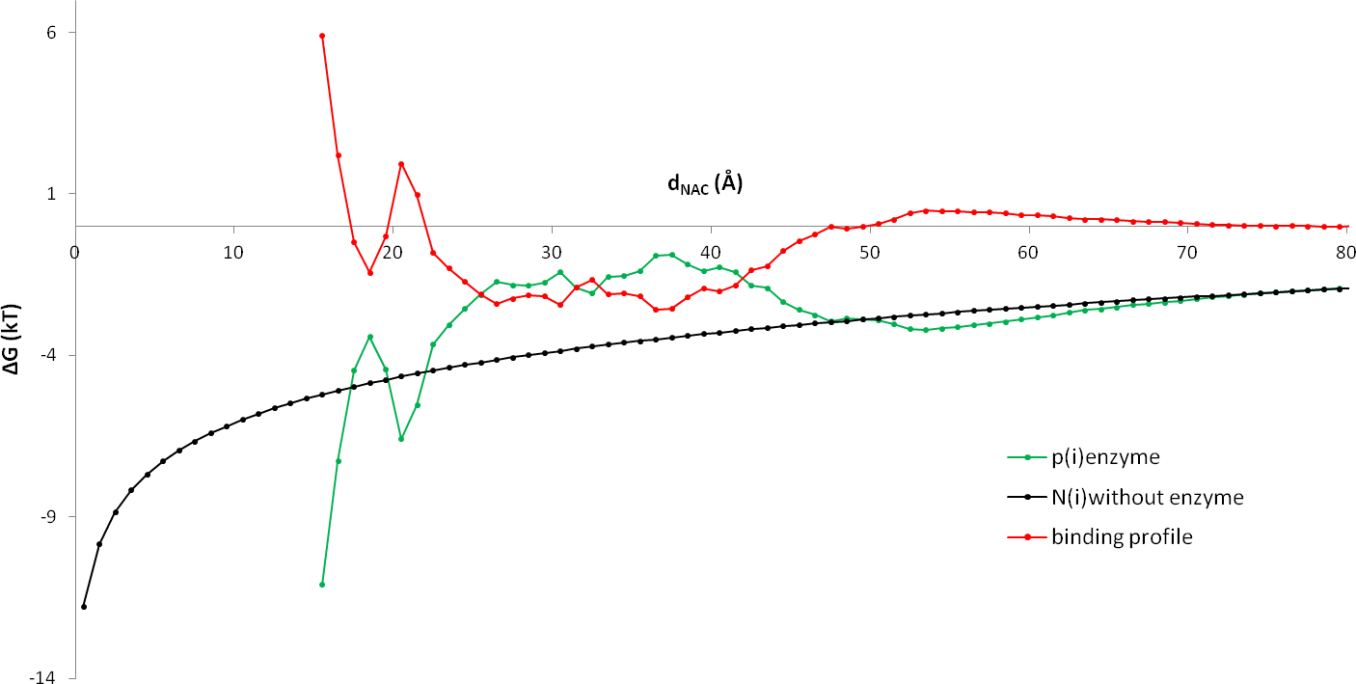


**Figure S3.** Binding profile of CPC binding to CA. The green line represents the probability of a CPC molecule at a given *d_NAC_* represented in logarithmic scale and corresponds to an energy (in kT). The black line represents the probability of a CPC molecule at a given *d_NAC_* without the enzyme in logarithmic scale. The red line is the binding profile: the difference between the black and the green curve.


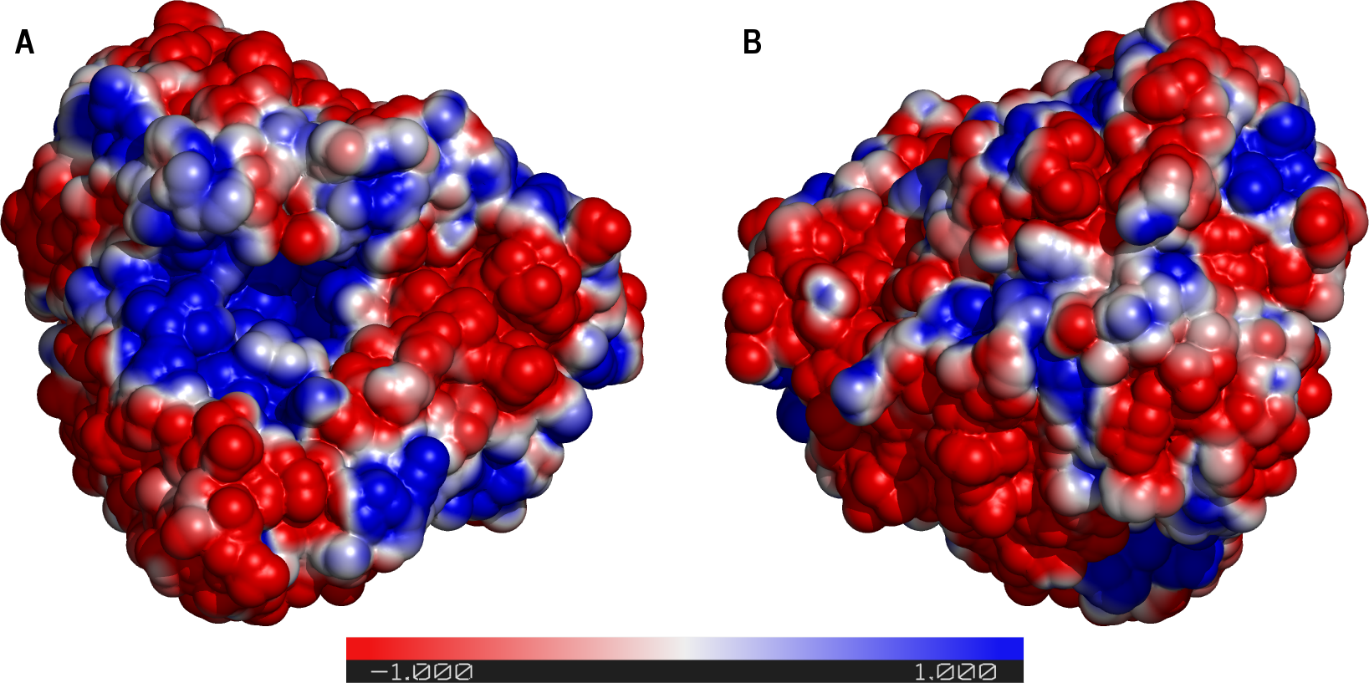


**Figure S4.** APBS analysis of the surface of WT. Negatively and positively charged patches are colored in red and blue, respectively (neutral patches in white). Front view with the positively charged entrance to the substrate binding pocket (A) and the back view of the protein, which is predominantly negatively charged (B)


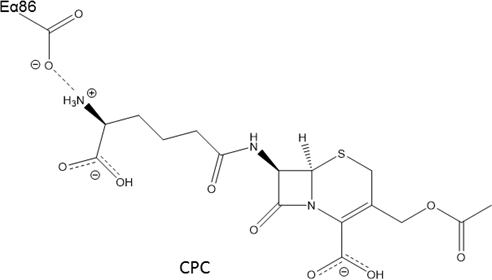


**Figure S5.** Structures of cephalosporin C (CPC) interacting with the side chain of E86α.


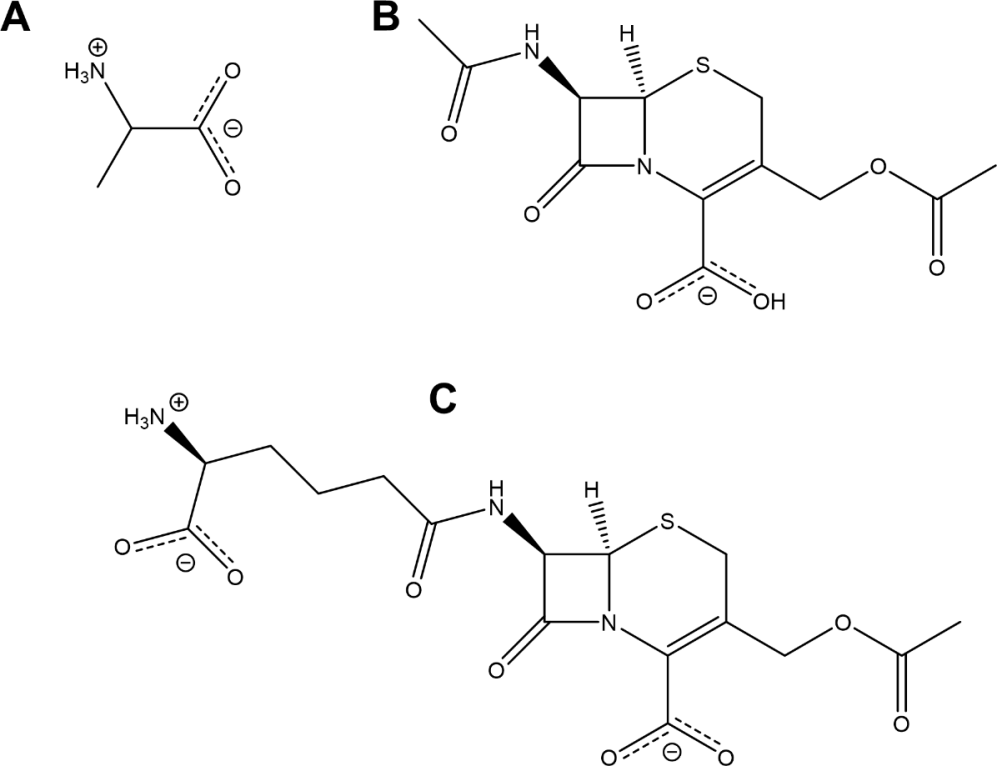


**Figure S6.** Schematic representation of the building block approach to obtain the CPC force field. The partial charges of the OPLS force field of alanine (A) were combined with the partial charges of the CPC core obtained from a RESP fit calculation (B). The complete CPC force field was obtained by generating the topology for standard atoms with the tool MKTOP and using the partial charges of the two building blocks A and B (C).

**Table S1.** Protonation of the histidines in the simulated protein models. For each histidine the position is indicated as well as the overall charge of the residue and the position of the hydrogen atoms defining the histidine model.

| **Protein chain** | **Histidine** | **Charge** | **Hydrogen position** |
| --- | --- | --- | --- |
| α | 43 | neutral | ε |
|  | 60 | neutral | ε |
|  | 153 | neutral | ε |
| β | 23 | neutral | ε |
|  | 35 | neutral | δ |
|  | 36 | neutral | ε |
|  | 57 | neutral | ε |
|  | 60 | neutral | δ |
|  | 70^*^ | neutral | δ |
|  | 76 | neutral | ε |
|  | 123 | neutral | δ |
|  | 178 | neutral | δ |
|  | 191 | neutral | δ |
|  | 216 | neutral | δ |
|  | 224 | neutral | δ |
|  | 250 | neutral | δ |
|  | 259 | neutral | ε |
|  | 286 | neutral | δ |
|  | 293 | neutral | δ |
|  | 359 | neutral | ε |
|  | 419 | neutral | δ |
|  | 425 | neutral | ε |
|  | 489 | neutral | δ |
|  | 494 | neutral | ε |
|  | 499 | neutral | δ |

**^*^ Residue 70 is histidine in the WT and it is mutated to serine in the M6 enzyme variant.**

**CPC force field definition used in the simulations**

[ moleculetype ]

; Name nrexcl

CLS 3

[ atoms ]

; nr type resnr residue atom cgnr charge mass typeB chargeB

1 opls_203 1 CLS S01 1 -0.30030 32.100000

2 opls_210 1 CLS C13 2 -0.00470 12.000000

3 opls_272 1 CLS C12 3 -0.04290 12.000000

4 opls_468 1 CLS C14 4 -0.01660 12.000000

5 opls_467 1 CLS O05 5 -0.40720 16.000000

6 opls_465 1 CLS C15 6 0.89860 12.000000

7 opls_466 1 CLS O16 7 -0.63290 16.000000

8 opls_135 1 CLS C01 8 -0.46240 12.000000

9 opls_145 1 CLS C10 9 0.03730 12.000000

10 opls_271 1 CLS C11 10 0.80300 12.000000

11 opls_272 1 CLS O13 11 -0.74610 16.000000

12 opls_272 1 CLS O12 12 -0.74610 16.000000

13 opls_239 1 CLS N03 13 -0.25020 14.000000

14 opls_211 1 CLS C09 14 0.10700 12.000000

15 opls_224B 1 CLS C07 15 -0.01920 12.000000

16 opls_235 1 CLS C08 16 0.58140 12.000000

17 opls_236 1 CLS O04 17 -0.51080 16.000000

18 opls_238 1 CLS N04 18 -0.52330 14.000000

19 opls_235 1 CLS C06 19 0.71650 12.000000

20 opls_236 1 CLS O02 20 -0.59150 16.000000

21 opls_136 1 CLS C16 21 -0.27340 12.000000

22 opls_136 1 CLS C17 22 -0.18000 12.000000

23 opls_136 1 CLS C18 23 -0.18000 12.000000

24 opls_299 1 CLS C19 24 0.15000 12.000000

25 opls_287 1 CLS N01 25 -0.30000 14.000000

26 opls_271 1 CLS C02 26 0.70000 12.000000

27 opls_272 1 CLS O01 27 -0.80000 16.000000

28 opls_272 1 CLS O03 28 -0.80000 16.000000

29 opls_140 1 CLS H15 29 0.06940 1.000000

30 opls_140 1 CLS H02 30 0.06940 1.000000

31 opls_140 1 CLS H09 31 0.09750 1.000000

32 opls_140 1 CLS H20 32 0.09750 1.000000

33 opls_140 1 CLS H03 33 0.11550 1.000000

34 opls_140 1 CLS H04 34 0.11550 1.000000

35 opls_140 1 CLS H05 35 0.11550 1.000000

36 opls_140 1 CLS H01 36 0.08560 1.000000

37 opls_140 1 CLS H12 37 0.10220 1.000000

38 opls_241 1 CLS H14 38 0.29070 1.000000

39 opls_140 1 CLS H21 39 0.11250 1.000000

40 opls_140 1 CLS H22 40 0.11250 1.000000

41 opls_140 1 CLS H19 41 0.09000 1.000000

42 opls_140 1 CLS H24 42 0.09000 1.000000

43 opls_140 1 CLS H06 43 0.09000 1.000000

44 opls_140 1 CLS H25 44 0.09000 1.000000

45 opls_140 1 CLS H07 45 0.06000 1.000000

46 opls_290 1 CLS H08 46 0.33000 1.000000

47 opls_290 1 CLS H11 47 0.33000 1.000000

48 opls_290 1 CLS H13 48 0.33000 1.000000

[ bonds ]

; ai aj funct r k

2 29 1 1.0930e-01 2.8108e+05

2 30 1 1.0930e-01 2.8108e+05

4 31 1 1.0930e-01 2.8108e+05

4 32 1 1.0930e-01 2.8108e+05

8 33 1 1.0920e-01 2.8225e+05

8 34 1 1.0920e-01 2.8225e+05

8 35 1 1.0920e-01 2.8225e+05

14 36 1 1.1000e-01 2.7313e+05

15 37 1 1.0970e-01 2.7648e+05

18 38 1 1.0090e-01 3.4326e+05

21 39 1 1.0920e-01 2.8225e+05

21 40 1 1.0920e-01 2.8225e+05

22 41 1 1.0920e-01 2.8225e+05

22 42 1 1.0920e-01 2.8225e+05

23 43 1 1.0920e-01 2.8225e+05

23 44 1 1.0920e-01 2.8225e+05

1 2 1 1.8210e-01 1.8895e+05

1 14 1 1.8210e-01 1.8895e+05

2 3 1 1.5080e-01 2.7472e+05

3 4 1 1.5080e-01 2.7472e+05

3 9 1 1.3390e-01 4.6903e+05

4 5 1 1.4390e-01 2.5230e+05

5 6 1 1.3430e-01 3.4418e+05

6 7 1 1.2140e-01 5.4225e+05

6 8 1 1.5080e-01 2.7472e+05

9 10 1 1.4740e-01 3.0443e+05

9 13 1 1.4070e-01 3.2677e+05

10 11 1 1.2140e-01 5.4225e+05

10 12 1 1.2140e-01 5.4225e+05

13 14 1 1.4600e-01 2.7665e+05

13 16 1 1.3450e-01 4.0016e+05

14 15 1 1.5540e-01 2.3999e+05

15 16 1 1.5290e-01 2.5815e+05

15 18 1 1.4600e-01 2.7665e+05

16 17 1 1.2140e-01 5.4225e+05

18 19 1 1.3450e-01 4.0016e+05

19 20 1 1.2140e-01 5.4225e+05

19 21 1 1.5080e-01 2.7472e+05

21 22 1 1.4990e-01 2.8225e+05

22 23 1 1.4990e-01 2.8225e+05

23 24 1 1.4990e-01 2.8225e+05

24 25 1 1.4630e-01 2.8200e+05

24 26 1 1.4990e-01 2.8225e+05

26 27 1 1.2140e-01 5.4225e+05

26 28 1 1.2140e-01 5.4225e+05

25 48 1

25 46 1

25 47 1

24 45 1

[ pairs ]

; ai aj funct

1 37 1

2 36 1

2 31 1

2 32 1

4 29 1

4 30 1

5 33 1

5 34 1

5 35 1

6 31 1

6 32 1

7 33 1

7 34 1

7 35 1

9 29 1

9 30 1

9 31 1

9 32 1

9 36 1

13 37 1

14 29 1

14 30 1

14 38 1

16 36 1

16 38 1

17 37 1

18 36 1

18 39 1

18 40 1

19 37 1

20 38 1

20 39 1

20 40 1

21 43 1

21 44 1

22 25 1

22 45 1

23 39 1

23 40 1

25 43 1

25 44 1

25 27 1

25 28 1

26 43 1

26 44 1

26 46 1

26 47 1

27 45 1

28 45 1

36 37 1

37 38 1

39 41 1

39 42 1

40 41 1

40 42 1

41 43 1

41 44 1

42 43 1

42 44 1

43 45 1

44 45 1

1 4 1

1 9 1

1 16 1

1 18 1

2 13 1

2 15 1

2 5 1

2 10 1

14 3 1

3 6 1

3 11 1

3 12 1

3 16 1

4 10 1

4 13 1

4 7 1

4 8 1

5 9 1

9 15 1

9 17 1

10 14 1

10 16 1

11 13 1

12 13 1

13 18 1

14 17 1

14 19 1

15 20 1

15 21 1

16 19 1

17 18 1

18 22 1

19 23 1

20 22 1

21 24 1

22 25 1

22 26 1

23 27 1

23 28 1

23 46 1

23 47 1

[ angles ]

; ai aj ak funct theta cth

1 2 29 1 1.0934e+02 4.4903e+02

1 2 30 1 1.0934e+02 4.4903e+02

1 14 36 1 1.0975e+02 3.5397e+02

3 2 29 1 1.1046e+02 3.9355e+02

3 2 30 1 1.1046e+02 3.9355e+02

3 4 31 1 1.1046e+02 3.9355e+02

3 4 32 1 1.1046e+02 3.9355e+02

5 4 31 1 1.0882e+02 4.2543e+02

5 4 32 1 1.0882e+02 4.2543e+02

6 8 33 1 1.0968e+02 3.9497e+02

6 8 34 1 1.0968e+02 3.9497e+02

6 8 35 1 1.0968e+02 3.9497e+02

13 14 36 1 1.0831e+02 4.1840e+02

14 15 37 1 1.1484e+02 3.7547e+02

15 14 36 1 1.1514e+02 3.7489e+02

15 18 38 1 1.1678e+02 3.8493e+02

16 15 37 1 1.0766e+02 3.9832e+02

18 15 37 1 1.0932e+02 4.1673e+02

19 18 38 1 1.1846e+02 4.1179e+02

19 21 39 1 1.0968e+02 3.9497e+02

19 21 40 1 1.0968e+02 3.9497e+02

22 21 39 1 1.1086e+02 3.9497e+02

22 21 40 1 1.1086e+02 3.9497e+02

21 22 41 1

21 22 42 1

22 21 39 1

22 21 40 1

22 23 43 1

22 23 44 1

23 22 41 1

23 22 42 1

23 24 45 1

24 23 43 1

24 23 44 1

24 25 46 1

24 25 47 1

25 24 45 1 1.0950e+02 4.1840e+02

26 24 45 1

29 2 30 1 1.0955e+02 3.2786e+02

31 4 32 1 1.0955e+02 3.2786e+02

33 8 34 1 1.0835e+02 3.2995e+02

33 8 35 1 1.0835e+02 3.2995e+02

34 8 35 1 1.0835e+02 3.2995e+02

39 21 40 1 1.0835e+02 3.2995e+02

41 22 42 1

43 23 44 1

46 25 47 1

1 2 3 1 1.0497e+02 6.7379e+02

1 14 13 1 1.1142e+02 5.3318e+02

1 14 15 1 1.1269e+02 5.1128e+02

2 1 14 1 9.9920e+01 5.0710e+02

2 3 4 1 1.1652e+02 5.2467e+02

2 3 9 1 1.2302e+02 5.3714e+02

3 4 5 1 1.0848e+02 5.7279e+02

3 9 10 1 1.2042e+02 5.5078e+02

3 9 13 1 1.2345e+02 5.7572e+02

4 3 9 1 1.2302e+02 5.3714e+02

4 5 6 1 1.1514e+02 5.3246e+02

5 6 7 1 1.2333e+02 6.3538e+02

5 6 8 1 1.1196e+02 5.7957e+02

7 6 8 1 1.2311e+02 5.6928e+02

9 10 11 1 1.2292e+02 5.7965e+02

9 10 12 1 1.2292e+02 5.7965e+02

9 13 14 1 1.2387e+02 5.1965e+02

9 13 16 1 1.2215e+02 5.4476e+02

10 9 13 1 1.2345e+02 5.7572e+02

11 10 12 1 1.3038e+02 6.5413e+02

13 14 15 1 1.1213e+02 5.4576e+02

13 16 15 1 1.0113e+02 5.6584e+02

13 16 17 1 1.2203e+02 6.3455e+02

14 13 16 1 1.2135e+02 5.3472e+02

14 15 16 1 8.4990e+01 6.0091e+02

14 15 18 1 1.1213e+02 5.4576e+02

15 16 17 1 1.3504e+02 5.3781e+02

15 18 19 1 1.2135e+02 5.3472e+02

16 15 18 1 1.1156e+02 5.5815e+02

18 19 20 1 1.2203e+02 6.3455e+02

18 19 21 1 1.1515e+02 5.6785e+02

19 21 22 1 1.1047e+02 5.4034e+02

20 19 21 1 1.2311e+02 5.6928e+02

21 22 23 1

22 23 24 1

23 24 25 1

24 26 27 1

24 26 28 1

25 24 26 1 1.1010e+02 5.2718e+02

27 26 28 1 1.3038e+02 6.5413e+02

24 25 48 1

46 25 48 1

47 25 48 1

[ dihedrals ]

;i j k l func C0 ... C5

1 14 15 37 3 0.65270 1.95811 0.00000 -2.61082 0.00000 0.00000 ;

2 1 14 36 3 1.39327 4.17982 0.00000 -5.57309 0.00000 0.00000 ;

2 3 4 31 3 0.00000 0.00000 0.00000 0.00000 0.00000 0.00000 ;

2 3 4 32 3 0.00000 0.00000 0.00000 0.00000 0.00000 0.00000 ;

4 3 2 29 3 0.00000 0.00000 0.00000 0.00000 0.00000 0.00000 ;

4 3 2 30 3 0.00000 0.00000 0.00000 0.00000 0.00000 0.00000 ;

5 6 8 33 3 0.00000 0.00000 0.00000 0.00000 0.00000 0.00000 ;

5 6 8 34 3 0.00000 0.00000 0.00000 0.00000 0.00000 0.00000 ;

5 6 8 35 3 0.00000 0.00000 0.00000 0.00000 0.00000 0.00000 ;

6 5 4 31 3 1.60247 4.80742 0.00000 -6.40989 0.00000 0.00000 ;

6 5 4 32 3 1.60247 4.80742 0.00000 -6.40989 0.00000 0.00000 ;

7 6 8 33 3 3.68192 -4.35136 0.00000 1.33888 0.00000 0.00000 ;

7 6 8 34 3 3.68192 -4.35136 0.00000 1.33888 0.00000 0.00000 ;

7 6 8 35 3 3.68192 -4.35136 0.00000 1.33888 0.00000 0.00000 ;

9 3 2 29 3 0.00000 0.00000 0.00000 0.00000 0.00000 0.00000 ;

9 3 2 30 3 0.00000 0.00000 0.00000 0.00000 0.00000 0.00000 ;

9 3 4 31 3 0.00000 0.00000 0.00000 0.00000 0.00000 0.00000 ;

9 3 4 32 3 0.00000 0.00000 0.00000 0.00000 0.00000 0.00000 ;

9 13 14 36 3 0.00000 0.00000 0.00000 0.00000 0.00000 0.00000 ;

13 14 15 37 3 0.65270 1.95811 0.00000 -2.61082 0.00000 0.00000 ;

13 16 15 37 3 0.00000 0.00000 0.00000 0.00000 0.00000 0.00000 ;

14 1 2 29 3 1.39327 4.17982 0.00000 -5.57309 0.00000 0.00000 ;

14 1 2 30 3 1.39327 4.17982 0.00000 -5.57309 0.00000 0.00000 ;

14 15 18 38 3 0.00000 0.00000 0.00000 0.00000 0.00000 0.00000 ;

16 13 14 36 3 0.00000 0.00000 0.00000 0.00000 0.00000 0.00000 ;

16 15 14 36 3 0.65270 1.95811 0.00000 -2.61082 0.00000 0.00000 ;

16 15 18 38 3 0.00000 0.00000 0.00000 0.00000 0.00000 0.00000 ;

17 16 15 37 3 0.00000 0.00000 0.00000 0.00000 0.00000 0.00000 ;

18 15 14 36 3 0.65270 1.95811 0.00000 -2.61082 0.00000 0.00000 ;

18 19 21 39 3 0.00000 0.00000 0.00000 0.00000 0.00000 0.00000 ;

18 19 21 40 3 0.00000 0.00000 0.00000 0.00000 0.00000 0.00000 ;

19 18 15 37 3 0.00000 0.00000 0.00000 0.00000 0.00000 0.00000 ;

19 21 22 23 3

19 21 22 41 3

19 21 22 42 3

20 19 18 38 3 29.28800 -8.36800 -20.92000 0.00000 0.00000 0.00000 ;

20 19 21 39 3 3.68192 -4.35136 0.00000 1.33888 0.00000 0.00000 ;

20 19 21 40 3 3.68192 -4.35136 0.00000 1.33888 0.00000 0.00000 ;

20 19 21 22 3

21 22 23 24 3

21 22 23 43 3

21 22 23 44 3

21 19 18 38 3 20.92000 0.00000 -20.92000 0.00000 0.00000 0.00000 ;

22 23 24 25 3

22 23 24 26 3

22 23 24 45 3

23 24 25 46 3

23 24 25 47 3

23 24 26 27 3

23 24 26 28 3

23 22 21 39 3

23 22 21 40 3

24 23 22 41 3

24 23 22 42 3

25 24 26 27 3

25 24 26 28 3

25 24 23 43 3

25 24 23 44 3

26 24 25 46 3

26 24 25 47 3

26 24 23 43 3

26 24 23 44 3

36 14 15 37 3 0.65270 1.95811 0.00000 -2.61082 0.00000 0.00000 ;

37 15 18 38 3 0.00000 0.00000 0.00000 0.00000 0.00000 0.00000 ;

41 23 24 42 3 33.47200 0.00000 -33.47200 0.00000 0.00000 0.00000 ;

42 24 25 43 3 33.47200 0.00000 -33.47200 0.00000 0.00000 0.00000 ;

19 15 18 38 3 9.20480 0.00000 -9.20480 0.00000 0.00000 0.00000 ;

1 2 3 4 3 0.00000 0.00000 0.00000 0.00000 0.00000 0.00000 ;

1 2 3 9 3 0.00000 0.00000 0.00000 0.00000 0.00000 0.00000 ;

1 14 13 9 3 0.00000 0.00000 0.00000 0.00000 0.00000 0.00000 ;

1 14 13 16 3 0.00000 0.00000 0.00000 0.00000 0.00000 0.00000 ;

1 14 15 16 3 0.65270 1.95811 0.00000 -2.61082 0.00000 0.00000 ;

1 14 15 18 3 0.65270 1.95811 0.00000 -2.61082 0.00000 0.00000 ;

2 1 14 13 3 1.39327 4.17982 0.00000 -5.57309 0.00000 0.00000 ;

2 1 14 15 3 1.39327 4.17982 0.00000 -5.57309 0.00000 0.00000 ;

2 3 4 5 3 0.00000 0.00000 0.00000 0.00000 0.00000 0.00000 ;

2 3 9 10 3 55.64720 0.00000 -55.64720 0.00000 0.00000 0.00000 ;

2 3 9 13 3 55.64720 0.00000 -55.64720 0.00000 0.00000 0.00000 ;

14 1 2 3 3 1.39327 4.17982 0.00000 -5.57309 0.00000 0.00000 ;

3 4 5 6 3 1.60247 4.80742 0.00000 -6.40989 0.00000 0.00000 ;

3 9 10 11 3 18.20040 0.00000 -18.20040 0.00000 0.00000 0.00000 ;

3 9 10 12 3 18.20040 0.00000 -18.20040 0.00000 0.00000 0.00000 ;

3 9 13 14 3 5.43920 0.00000 -5.43920 0.00000 0.00000 0.00000 ;

3 9 13 16 3 5.43920 0.00000 -5.43920 0.00000 0.00000 0.00000 ;

4 3 9 10 3 55.64720 0.00000 -55.64720 0.00000 0.00000 0.00000 ;

4 3 9 13 3 55.64720 0.00000 -55.64720 0.00000 0.00000 0.00000 ;

4 5 6 7 3 28.45120 5.85760 -22.59360 0.00000 0.00000 0.00000 ;

4 5 6 8 3 22.59360 0.00000 -22.59360 0.00000 0.00000 0.00000 ;

5 4 3 9 3 0.00000 0.00000 0.00000 0.00000 0.00000 0.00000 ;

9 13 14 15 3 0.00000 0.00000 0.00000 0.00000 0.00000 0.00000 ;

9 13 16 15 3 20.92000 0.00000 -20.92000 0.00000 0.00000 0.00000 ;

9 13 16 17 3 20.92000 0.00000 -20.92000 0.00000 0.00000 0.00000 ;

10 9 13 14 3 5.43920 0.00000 -5.43920 0.00000 0.00000 0.00000 ;

10 9 13 16 3 5.43920 0.00000 -5.43920 0.00000 0.00000 0.00000 ;

11 10 9 13 3 18.20040 0.00000 -18.20040 0.00000 0.00000 0.00000 ;

12 10 9 13 3 18.20040 0.00000 -18.20040 0.00000 0.00000 0.00000 ;

13 14 15 16 3 0.65270 1.95811 0.00000 -2.61082 0.00000 0.00000 ;

13 14 15 18 3 0.65270 1.95811 0.00000 -2.61082 0.00000 0.00000 ;

13 16 15 14 3 0.00000 0.00000 0.00000 0.00000 0.00000 0.00000 ;

13 16 15 18 3 0.00000 0.00000 0.00000 0.00000 0.00000 0.00000 ;

14 13 16 15 3 20.92000 0.00000 -20.92000 0.00000 0.00000 0.00000 ;

14 13 16 17 3 20.92000 0.00000 -20.92000 0.00000 0.00000 0.00000 ;

14 15 16 17 3 0.00000 0.00000 0.00000 0.00000 0.00000 0.00000 ;

14 15 18 19 3 0.00000 0.00000 0.00000 0.00000 0.00000 0.00000 ;

15 14 13 16 3 0.00000 0.00000 0.00000 0.00000 0.00000 0.00000 ;

15 18 19 20 3 20.92000 0.00000 -20.92000 0.00000 0.00000 0.00000 ;

15 18 19 21 3 20.92000 0.00000 -20.92000 0.00000 0.00000 0.00000 ;

16 15 18 19 3 0.00000 0.00000 0.00000 0.00000 0.00000 0.00000 ;

17 16 15 18 3 0.00000 0.00000 0.00000 0.00000 0.00000 0.00000 ;

18 19 21 22 3 0.00000 0.00000 0.00000 0.00000 0.00000 0.00000 ;

19 21 22 23 3 0.00000 0.00000 0.00000 0.00000 0.00000 0.00000 ;

19 21 22 26 3 0.00000 0.00000 0.00000 0.00000 0.00000 0.00000 ;

20 19 21 22 3 0.00000 0.00000 0.00000 0.00000 0.00000 0.00000 ;

21 22 23 24 3 33.47200 0.00000 -33.47200 0.00000 0.00000 0.00000 ;

21 22 26 25 3 9.20480 0.00000 -9.20480 0.00000 0.00000 0.00000 ;

22 23 24 25 3 33.47200 0.00000 -33.47200 0.00000 0.00000 0.00000 ;

22 26 25 24 3 9.20480 0.00000 -9.20480 0.00000 0.00000 0.00000 ;

23 22 26 25 3 9.20480 0.00000 -9.20480 0.00000 0.00000 0.00000 ;

23 24 25 26 3 33.47200 0.00000 -33.47200 0.00000 0.00000 0.00000 ;

24 23 22 26 3 33.47200 0.00000 -33.47200 0.00000 0.00000 0.00000 ;

2 4 3 9 3 9.20480 0.00000 -9.20480 0.00000 0.00000 0.00000 ;

8 7 6 5 3 87.86400 0.00000 -87.86400 0.00000 0.00000 0.00000 ;

10 3 9 13 3 9.20480 0.00000 -9.20480 0.00000 0.00000 0.00000 ;

9 11 10 12 3 9.20480 0.00000 -9.20480 0.00000 0.00000 0.00000 ;

16 9 13 14 3 9.20480 0.00000 -9.20480 0.00000 0.00000 0.00000 ;

15 13 16 17 3 87.86400 0.00000 -87.86400 0.00000 0.00000 0.00000 ;

21 18 19 20 3 87.86400 0.00000 -87.86400 0.00000 0.00000 0.00000 ;

21 23 22 26 3 9.20480 0.00000 -9.20480 0.00000 0.00000 0.00000 ;

23 22 25 48 3

45 22 25 48 3
